# Editorial: Novel roles for tumor-associated neutrophils

**DOI:** 10.3389/fimmu.2022.1004772

**Published:** 2022-08-18

**Authors:** Susanna Mandruzzato, Heinz Läubli

**Affiliations:** ^1^ Department of Surgery, Oncology and Gastroenterology, University of Padova, Padova, Italy; ^2^ Immunology and Molecular Oncology Diagnostics, Veneto Institute of Oncology IOV – Istituto di Ricovero e Cura a Carattere Scientifico, Padova, Italy; ^3^ Department of Biomedicine, University of Basel, Basel, Switzerland; ^4^ Division of Medical Oncology, University Hospital Basel, Basel, Switzerland

**Keywords:** neutrophils (PMNs), cancer, tumor microenvironment, polymorphonuclear neutrophil, neutrophil extracellular trap

In recent years, the importance of the activity of neutrophils during cancer progression has been highlighted by many studies. The impact of neutrophils on tumor initiation and progression can be twofold, as they can have both protumor and antitumor effects, and their influence can be exerted within the tumor microenvironment as well as systemically.

This Research Topic collects results evaluating the role of neutrophils ranging from pre-metastatic tumors in preclinical models, to hematological and solid human tumors, and to a bioinformatic approach to analyze the signature of a phenomenon set in motion by neutrophils in a large cohort of solid tumors.

Neutrophils release several molecules, including DNA, and the resulting protein-covered DNA structures are called neutrophils extracellular traps (NET), which have been implicated in protumoral activity. In this regard, the original article by Shen et al. performed the analysis of NET profiles that were downloaded from TCGA in about 8000 cancer patients across 22 major human cancers. A pan-cancer NET signature of 23 genes was identified for each cancer that was associated to the major hallmarks of cancer, including the inflammatory response that showed the highest correlation. Furthermore, the correlation between NETs and patient’s prognosis was investigated, and distinct survival patterns emerged, depending on the tumor type analyzed.

NETs released by immature and mature neutrophils have been implicated also in damaging the integrity of endothelial cells. In their original article Wang et al. explore the role of NETs in acute promyelocytic leukemia (APL) in which NETosis is involved. Results indicate that immature neutrophils have a reduced ability to induce NETs, and that all-trans retinoic acid (ATRA) with arsenic trioxide therapy used for these patients induced an increased in NETs from mature neutrophils, thus sustaining the hypothesis that the excessive NETs damage endothelial cells, causing blood cell leakage, which may explain the poorly understood phenomenon of hemorrhage in APL patients under treatment.

Yang et al. evaluate the clinical significance of tumor-associated neutrophils (TAN) in the tumor microenvironment of urothelial carcinoma of the bladder (UCB). More specifically, authors evaluate 237 cases of resectable UCB and identify the density of neutrophils in the stromal region as an independent prognostic factor for overall survival (OS). In addition, a number of evidences indicate that neutrophils might play an immunosuppressive role on T cell immunity partially *via* PD-L1.

The study by SenGupta et al. investigate the function of TAN in triple-negative breast cancer (TNBC). In particular, the authors analyzed how TAN are migrating to the tumor microenvironment by *in vitro* models. They find an increased migratory potential of TAN toward highly aggressive TNBC cells compared to cells from other types of breast cancer. Furthermore, the investigators identify chemokines including CXCL-1 as responsible factor for the increased migratory potential. Blockade of identified chemokines is able to abrogate chemoattraction of neutrophils.

The role of neutrophils in the pre-metastatic tumor is evaluated by Hussain et al., in tumor-draining lymph nodes (TDLNs) of a mouse model of head and neck cancer (HNC). Immune responses initiated in TDLNs have the potential to block or to support tumor growth and to evaluate the role of neutrophils in this context, authors investigate mice deficient in type I interferon (IFN), a cytokine promoting the anti-tumoral activity of neutrophils. Accordingly, deficient mice show elevated tumor growth and metastatic spread, and the mechanisms of defective type I interferon signaling on the activity of neutrophils in TDLNs are analyzed by live imaging and by phenotypic and functional analyses.

Finally, the review by Lin et al. discusses the origin and functions of neutrophils in the context of gliomas and brain metastases. The presence and the role of TANs is discussed in the complex and unique scenario of the tumor microenvironment (TME) of brain tumors, along with the potential therapeutic implications of neutrophils as carriers of drugs to the brain TME.

In conclusion, the present Research Topic demonstrates a strong and important research activity on the function of neutrophils in cancer. Although it has become evident that neutrophils play an important role in cancer initiation and progression, further investigations are needed to identify therapeutic targets to improve cancer therapy by interfering with TAN. These six excellent studies provide a basis for potential new approaches to target TAN for cancer therapy.

## Author contributions

All authors have made a substantial and intellectual contribution to the work and approved it for publication.

## Conflict of interest

The authors declare that the research was conducted in the absence of any commercial or financial relationships that could be construed as a potential conflict of interest.

## Publisher’s note

All claims expressed in this article are solely those of the authors and do not necessarily represent those of their affiliated organizations, or those of the publisher, the editors and the reviewers. Any product that may be evaluated in this article, or claim that may be made by its manufacturer, is not guaranteed or endorsed by the publisher.

